# Wastewater Treatment: Functional Materials and Advanced Technology

**DOI:** 10.3390/molecules29092150

**Published:** 2024-05-06

**Authors:** Jingtao Bi, Guohui Dong

**Affiliations:** 1 Engineering Research Center of Seawater Utilization of Ministry of Education, School of Chemical Engineering and Technology, Hebei University of Technology, Tianjin 300401, China; 2 School of Environmental Science and Engineering, Shaanxi University of Science and Technology, Xi’an 710021, China

With accelerated advancements in various industries, water pollution has emerged as a significant issue characterized by two features: (1) the rapid increase in population and corresponding demands, leading to a sharp rise in wastewater discharge, and (2) the development of new technologies, contributing to a significant increase in the variety of emerging contaminants, resulting in a more complex wastewater composition [1,2,3,4]. Against this background, we proposed and launched a Special Issue, “Wastewater Treatment: Functional Materials and Advanced Technology”, to provide feasible methods and insights for addressing these two features.

According to the publication status of the Special Issue, 34 publications have been released, including 4 reviews, 2 communications, and 28 full-length research articles. Focused on the theme of the Special Issue, the publications have reported a series of functional adsorption materials, ion sieves, and oxidation/reduction materials for typical contaminants such as salts, heavy metals, radioactive nuclides, soluble organic pollutants, and oils. Additionally, they have reported on related technologies such as adsorption, ion exchange, oxidation, reduction, oil–water separation, reverse osmosis, and electrodialysis. To deepen readers’ understanding of this Special Issue, we have also created a word cloud based on the keywords of the 34 papers in the Special Issue, as shown in Figure 1.

Overall, “Wastewater Treatment: Functional Materials and Advanced Technology” has achieved certain results. However, considering the current trends in wastewater treatment research and the status of publications in this Special Issue, we consider that future research could focus on the following aspects, which will be welcomed in the second edition of this Special Issue:

(1) Wastewater treatment is application-oriented [5] and should focus on existing wastewater with specific treatment challenges. Therefore, while emphasizing theoretical innovation, exploring the application of relevant materials and technologies in real wastewater and specific application scenarios is particularly important.

(2) Emerging pollutants present new challenges for wastewater treatment [6,7]. Efficient treatment materials and technologies for emerging pollutants, such as microplastics, radionuclides, new drugs and their metabolites, and per/polyfluoroalkyl substances (PFASs), are particularly crucial.

(3) Process intensification has wide applications in wastewater treatment [8,9,10]. Some articles in this Special Issue have discussed ultrasonic intensification in materials fabrication and treatment technology [11,12]. To further enhance research in this area, emerging techniques such as nanoscale confinement intensification and external field (electric field, magnetic field, gravity field, etc.) intensification technologies are frontier topics worthy of attention.

(4) Although many efficient functional materials and advanced processes have been reported for wastewater treatment, their economic balance has received less investigation. Wastewater treatment is often considered a “rescue” step in various processes, with cost remaining at its core. Therefore, the focus should also be on the economy while considering treatment efficiency.

## Figures and Tables

**Figure 1:**
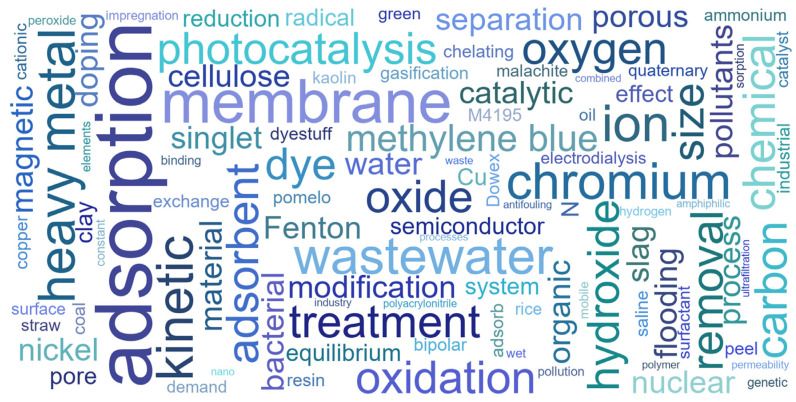
Keyword cloud of the 34 papers in the Special Issue.
